# *Ascaridia pavo* n. sp. (Nematoda: Ascaridida), a new species of nematode in peafowl

**DOI:** 10.1590/S1984-29612026006

**Published:** 2026-05-25

**Authors:** Raul Henrique da Silva Pinheiro, Roberto Rafael de Queiroz Pereira Rodrigues, Raimundo Nonato Moraes Benigno, Luis Augusto Araújo dos Santos Ruffeil, Elane Guerreiro Giese

**Affiliations:** 1 Universidade Federal Rural da Amazônia – UFRA, Instituto da Saúde e Produção Animal, Laboratório de Histologia e Embriologia Animal, Belém, PA, Brasil; 2 Médico Veterinário autônomo, Belém, PA, Brasil; 3 Universidade Federal Rural da Amazônia – UFRA, Instituto da Saúde e Produção Animal, Laboratório de Parasitologia Animal, Belém, PA, Brasil; 4 Escola Estadual de Ensino Técnico do Pará Dr. Celso Malcher, Belém, PA, Brasil

**Keywords:** Taxonomy, nematode, parasite, birds, Amazon, Taxonomia, nematoda, parasito, aves, Amazônia

## Abstract

A new Ascaridiidae has been found parasitizing *Pavo cristatus*, an exotic bird bred on a commercial farm in the Pará Amazon. The new species of *Ascaridia* Dujardin, 1845 is described on the basis of light and scanning microscopy findings. Morphologically, the new species shares characteristics compatible with the other species of *Ascaridia* that parasitize birds in Brazil, however, the new species differs from *A. pintoi* and *A. ornata* in the length and absence of a wing on the spicules, *A. hermaphrodita*, *A. serrata*, *A. columbae*, *A. galli*, *A. lineata*, *A. magalhaesi*, *A. numidae*, *A. orthocerca*, *A. pterophora* and *A. sergiomeirai* in the distribution and number of caudal papillae in males, *A. amblymoria* in the presence of a single papilla on the upper lip, associated with these characteristics most species differ in host order, with only *A. columbae*, *A. galli* and *A. lineata*, parasites of birds in the Phasianidae family. The new taxon adds morphological data to the parasitic biodiversity of commercially reared birds in Brazil.

## Resumo

Um novo Ascaridiidae foi encontrado parasitando *Pavo cristatus*, ave exótica criada em uma granja comercial na Amazônia paraense. A nova espécie de *Ascaridia* Dujardin, 1845 é descrita com base em achados de microscopia de luz e varredura. Morfologicamente, a nova espécie partilha características compatíveis com as demais espécies de *Ascaridia* que parasitam aves no Brasil, porém, a nova espécie difere de *A. pintoi* e *A. ornata* no comprimento e ausência de uma asa nos espiculos, *A. hermafrodita*, *A. serrata*, *A. columbae*, *A. galli*, *A. lineata*, *A. magalhaesi*, *A. numidae*, *A. orthocerca*, *A. pterophora* e *A. sergiomeirai* na distribuição e no número de papilas caudais nos machos, *A. amblymoria* na presença de uma papila única no lábio superior, associados a essas características a maioria das espécies diferem na ordem dos hospedeiros, sendo apenas *A. columbae*, *A. galli* e *A. lineata*, parasitos de aves da família Phasianidae. O novo táxon contribui com adição de dados morfológicos à biodiversidade parasitária de aves criadas comercialmente no Brasil.

## Introduction

Brazil has a rich avifauna, and parasitological studies in its birds began with samples collected by Johann Natterer when he carried out 16 scientific expeditions in the interior of Brazil, starting in Rio de Janeiro and heading towards Mato Grosso and then to the Amazon basin, as well as Colombia and Venezuela (Goeldi, 1896; Vanzolini, 1993). Karl Asmund Rudolphi, Karl Moriz Diesing, Raffaele Molin and Anton Friedrich Schneider, when analyzing the samples collected by Natterer, described the first helminth parasites of birds in Brazil. However, the descriptions are in the main very poorly done, are composed of only a few lines and usually lack figures or drawings to support the study (Guerrero, 2021).

In 1912, Railliet & Henry proposed the creation of the subfamily Heterakinae to include the genera *Ascaridia* Dujardin, 1845; *Heterakis* Dujardin, 1845; *Subulura* Molin, 1860; *Aspidodera* Railliet & Henry, 1912 and *Cissophyllus* Railliet & Henry, 1912, morphologically identical in the presence of a pre-anal sucker, but throughout their taxonomic history the species of *Ascaridia* have been broken down into other genera, especially *Ascaris* Linnaeus, 1758 and *Heterakis* Dujardin, 1886, with the type species *Ascaridia hermaphrodita* (Froelich, 1789) Railliet & Henry, 1914, being relocated from the genus *Ascaris* (*A. hermaphrodita* Froelich, 1789) (Travassos, 1913). Morphologically, *Ascaridia* has a claviform esophagus, while *Heterakis* has an esophageal bulb, and *Ascaris* has lips with rows of denticles, a distinct characteristic of the other two genera (Anderson et al., 2009).

*Ascaridia* is made up of more than 50 species and has a cosmopolitan distribution. They parasitize the intestines of different types of birds, especially terrestrial species (Yamaguti, 1961; Hodová et al., 2008). In Brazil, it is represented by *Ascaridia hermafrodita*; *Ascaridia serrata* (Schneider, 1866) Railliet & Henry, 1914; *Ascaridia amblymoria* (Drasche, 1882) Railliet & Henry, 1914; *Ascaridia columbae* (Gmelin, 1790) Travassos, 1913; *Ascaridia galli* (Schrank, 1788) Freeborn, 1923 (Syn. *Ascaridia brasiliensis* (Magalhães, 1892)); *Ascaridia lineata* (Schneider, 1866) Railliet & Henry, 1912; *Ascaridia magalhaesi* Travassos, 1913; *Ascaridia numidae* (Leiper, 1908) Travassos, 1913; *Ascaridia ornata* Kreis, 1955; *Ascaridia orthocerca* (Stossich, 1902) Travassos, 1913; *Ascaridia pintoi* Travassos, 1933; *Ascaridia pterophora* (Creplin, 1854) Railliet & Henry, 1914 and *Ascaridia sergiomeirai* Pereira, 1933, all parasites of wild and farmed birds (Vicente et al., 1995). *Ascaridia hermaphrodita* and *A. galli* are the two most reported species (Santos et al., 2015; Tavares et al., 2017; Ramos et al., 2018), in addition to the record of an erratic cycle of *A. galli* in eggs of *Gallus gallus domesticus* (Linnaeus, 1758) (Machado et al., 2007, Bautista-Vanegas et al., 2023) and unusual hosts for the genus such as *Felis catus* Linnaeus, 1758 (Duarte, 1981; Vicente et al., 1997) and *Cerdocyon thous* Linnaeus, 1766 (Gomes, 2013; Gomes et al., 2015) in Brazil and the presence of *Ascaridia* sp. eggs in feces in dogs in some urban and rural areas of Hungary (Fok et al., 2001).

The peacock is a bird of the Phasianidae family, native to Asia, found in India, Myanmar, Java Island and the Malay Peninsula. Its geographical distribution has broadened as it has been introduced into various countries, including Brazil, where it is bred along with other domestic birds (Ramos et al. 2018). For this study, nematodes were recovered from the intestine of a specimen of *Pavo cristatus* Linnaeus, 1758 (Aves: Galliformes: Phasianidae), and subsequently studied in detail, concluding that they represent a new species, which is described here.

## Material and Methods

A rural landowner with commercial breeding of *Pavo cristatus*, located in the municipality of Benevides in the state of Pará, in the Brazilian Amazon region. He obtained 12 of these birds from a cross between his breeding stock. During the first 5 months (average weight 2kg) of life, the animals were placed in a vivarium measuring 12m^2^ wide by 2m high with a stainless-steel mesh floor raised 1.5m off the ground and isolated from other birds on the property by a screen. The water was served in automatic drinking fountains, and growth feed was provided, compatible with their age.

Two birds died, both with good body scores. The animals were submitted to necropsy on the property; the intestines were separated and fixed in 10% formalin. After these were obtained, the owner administered fembendazole (Provermim®) (deworming), a packet of 20g for every 2kg of feed for 3 days in a row for the other birds, as well as sanitizing the environment.

In the laboratory, the intestine was separated and placed in Petri dishes with saline and examined using a stereomicroscope. When the intestines were examined, there were large numbers of nematodes all over the intestines, causing organ obstruction. The nematodes were removed, washed in 0.9% saline solution, quantified and processed for light microscopy and scanning electron microscopy, following the protocol described by Pinheiro et al. (2018).

All measurements are presented in millimeters, unless otherwise indicated. Morphometric information is presented as mean, standard deviation, ranges in parentheses, and holotype and allotype measurements in square brackets. The type material was deposited in the Coleção de Invertebrados não Artrópodes at the Museu Paraense Emílio Goeldi (MPEG), municipality of Belém, State of Pará, Brazil: Type-material: Holotype male (MPEG 319), allotype female (MPEG 320) and paratypes (five females: MPEG 321; five males: MPEG 322). Zoobank Life Science Identifier: urn:lsid:zoobank.org:pub:03DF4CF4-0EE5-42D2-989F-E349BD91B23A

## Results

*Ascaridia* Dujardin, 1844

**Figure 1:**
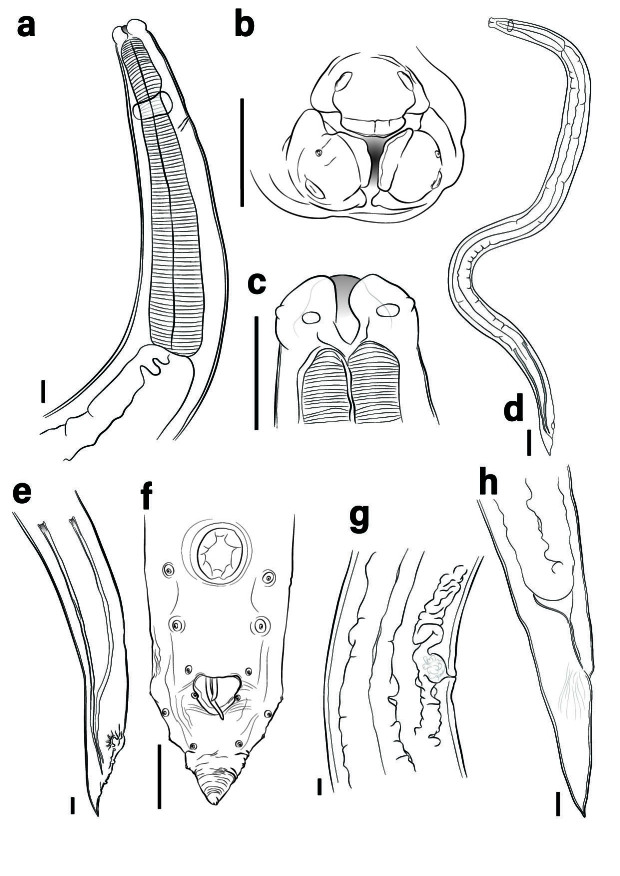
*Ascaridia pavo***n. sp.** from *Pavo cristatus*: (**a**) Anterior extremity of female, lateral view. Scale bars = 100µm. (**b**) Cephalic region, apical view. Scale bars = 100µm. (**c**) Cephalic region of male, lateral view. Scale bars = 100µm. (**d**) Male, total length. Scale bars = 500µm. (**e, f**) Posterior end of male, lateral and ventral views, respectively. Scale bars = 100µm. (**g**) Vulva. Scale bars = 100µm. (**h**) Posterior end of female, lateral view. Scale bars = 100µm.

**Figure 2:**
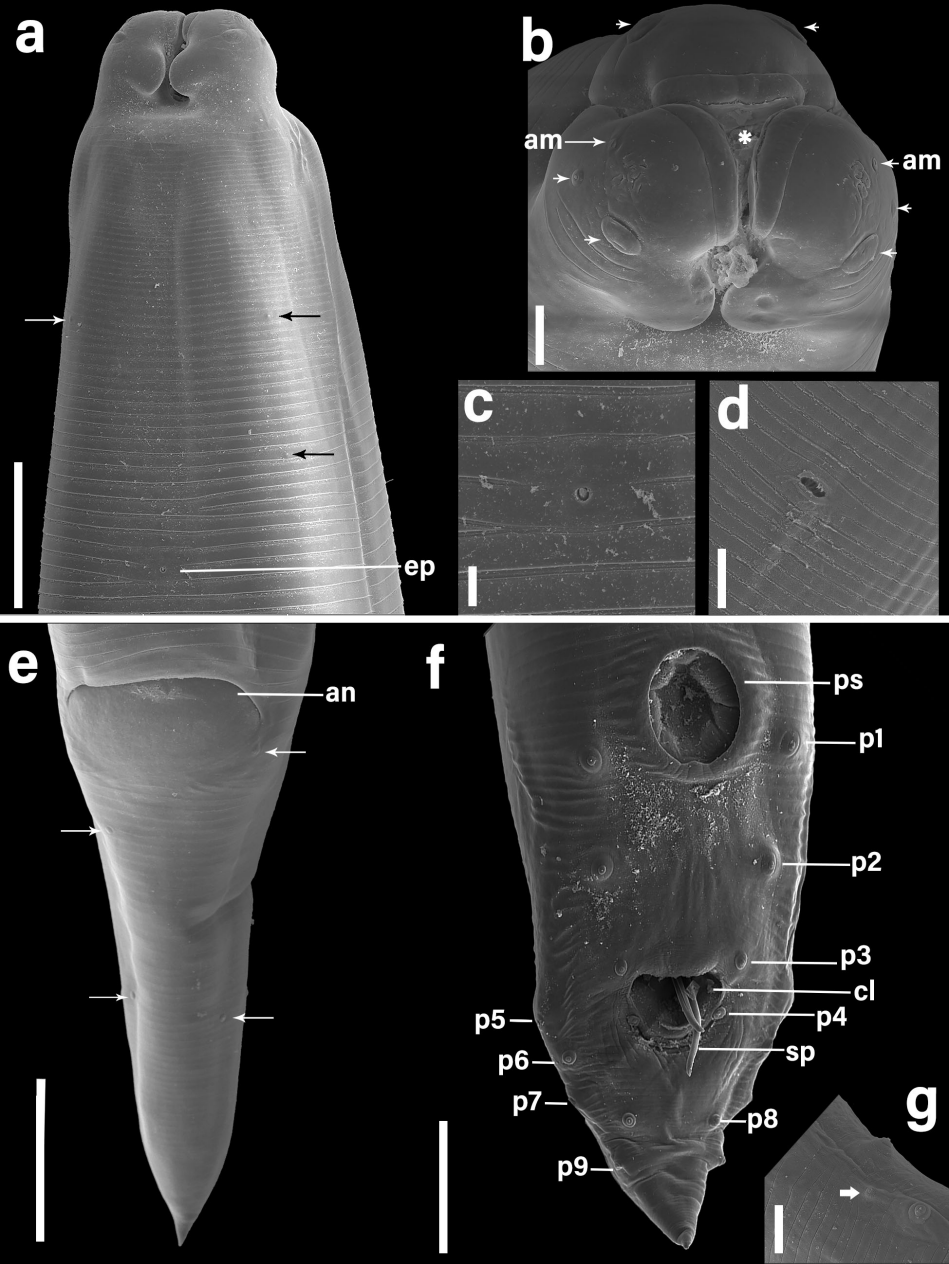
Scanning electron micrographs of *Ascaridia pavo* **n. sp.** from *Pavo cristatus*: (**a)** Cephalic region, ventral view, with excretory pore (ep) and presence of small papillae (arrow). Scale bars = 10μm. **(b)** Cephalic region, apical view, with 3 trilobed lips, with prominent lateral membranous flanges, dorsal lip with a pair of large double papillae; each of the ventral-lateral lips with a large papilla, a small papilla (arrow) and an amphid (am). Inner surface of the distal margin of each lip with a cuticular plate which at the tip forms tooth-like projections. Scale bars = 10μm. (**c**) Excretory pore. Scale bars = 20μm. (**d)** Vulva, vulval lips not elevated. Scale bars = 20μm. (**e**) Female tail, anus (an) and presence of small papillae (arrow). Scale bars = 50μm. (**f**) Tail of male, ventral views; cloacal opening, spicule, three precloacal papillae (p1, p2, p3), one pair of papillae situated paracloacally (p4), five postcloacal papillae, latter arranged as: p5, p7 and p9 pairs laterally, p6 pair located subventrally and p8 pair located ventrally. Scale bars = 100μm. (**g**) One pair of phasmids lateral. Scale bars = 20μm.

***Ascaridia pavo* n. sp.** (Figures 1-2)

*Etymology:* The specific name refers to the generic name of the type host.

### Description

General. Medium to large, whitish nematodes, with distinct cuticle. Lateral alae absent. Anterior extremity with 3 trilobed lips, approximately equal in size, with weak postlabial grooves and prominent lateral membranous flanges (Figures 1a, b, 2a). Dorsal lip with a pair of large double papillae; each ventral-lateral lip having one large double papilla, one small papilla and an amphid (Figure 1c, 2b). Inner surface of distal margin of each lip with several cuticular plates that form tooth-like projections at the tip. Interlabia absent. Esophagus cylindrical and slightly extended towards the posterior end without a posterior bulb (Figure 1a). Nerve-ring at about 14−39% of esophageal length. Excretory pore posterior to nerve-ring (Figure 2a, c). Ventriculus, intestinal caecum and ventricular appendix absent. Small papillae distributed randomly along the body (Figure 2a).

**Males (based on 15 specimens all adult, measurements of holotype in brackets)**: Body length 14.00±14.00 (8.00–22.00) [14.00], maximum width 0.521±135.00 (0.353–0.740) [0.547] at level of esophageal-intestinal junction (Figure 1d). Dorsal and ventral-lateral lips almost equal in size, 0.111±0.020 (0.083–0.137) [0.137] long, 0.163±0.027 (0.127–0.190) [0190] wide. Nerve ring and excretory pore 0.404±92.00 (0.307–0.587) [0.367] and 0.509±100.00 (0.413–0.693) [0.427], in lateral view respectively, from anterior extremity. Muscular esophagus 2.00±0.00 (1.00–2.00) [1.00] long, 0.255±66.00 (0.200–0.400) [0.240] at maximum width, representing 11% (9–15%) [9.7%] of body length. Posterior end of body curves ventrally. Ventral sucker present with smooth adhesive surface, situated at 0.435±150.00 (0.173–0.633) [0.533] from cloacal aperture. Caudal papillae, 9 pairs in all, arranged as follows: 3 pairs precloacal (1^st^ pair at the level of the sucker, 2^nd^ pair posterior to the sucker, 3^rd^ near the cloaca.), 4^th^ pair paracloacal (located laterally at level to cloaca) and 5 pairs postcloacal; latter arranged as: 5^th^, 7^th^ and 9^th^ pairs laterally, 6^th^ pair located subventrally and 8^th^ pair located ventrally (Figures 1f, 2f), one pair of lateral phasmids, between the 7^th^ and 9^th^ pairs of postcloacal papillae (Figure 2g). All the papillae are mammillae-shaped and arise from two circles of cuticular rings, with a knob at the tip, the 1^st^ and 2^nd^ precloacal pairs larger than the others (Figure 2f). Medio-ventral precloacal papilla and cuticular bosses absent. Spicules long, filiform, sub-equal and sclerotized 2.00±0.00 (1.00–3.00) [2.00] long, representing 14% (10–21%) of total body length (Figure 1e). Gubernaculum absent. Tail 0.268±51.00 (0.187–0.367) [0.240] long.

**Females (based on 10 specimens all gravid with eggs, measurements of allotype in brackets)**: Body 17.00±3.00 (14.00–24.00) [16.71] long; maximum width 0.549±71.60 (0.407–0.653) [0.607]. Dorsal and ventral-lateral lips almost equal in size, 0.106±0.004 (0.100–0.110) [0.100] long, 0.147±0.012 (0.130–0.157) [0.155] wide. Nerve ring and excretory pore 0.345±92.95 (0.260–0.507) [0.507] and 0.481±72.00 (0.347–0.607) [0.580], in lateral view respectively, from anterior extremity. Muscular esophagus 2.00±0.2 (1.00–2.00) [1.67] long, 0.241±28.58 (0.187–0.300) [0.240] in maximum width, representing 9% (4–11%) [10%] of body length. Slitlike vulva, situated at mid-body, 9.00±1.5 (8.00–13.00) [9.71] mm from anterior extremity, at 55% (53–59%) [58%] of body length (Figures 1g, 2d). Vagina muscular, directed posteriorly from vulva, vulval lips not elevated. Two uteri, one in anterior part of body, the other in posterior part. Eggs oval, unembryonated, with smooth shell, 0.064±9.63 (0.048–0.087) × 0.043±7.46 (0.022–0.057) (n = 30). Rectum is a short hyaline tube; small, unicellular rectal glands are present measuring 0.333±69.00 (0.200‒0.407) [0.367]. Tail 0.604±63.00 (0.467–0.687) [0.560] long (Figures 1h, 2e).

## Discussion

From the descriptions of *Ascaridia* species, it was evident that many morphological features were no longer present. The most effective strategies for grouping and comparing species proved to be the host group parasitized by the species (mainly at the host order level); intraspecific variability, probably resulting from the presence of the parasites in different hosts; the intensity of infection; and geographical location (Zhao et al., 2016). With that in mind, to avoid describing a species that might be synonymized in the future, in this paper we will compare the new taxon with all the known species of the genus in Brazil.

In Brazil, 13 species of *Ascaridia* have been recorded parasitizing different groups of birds (Vicente et al., 1995). *Ascaridia pavo* n. sp. has 9 pairs of caudal papillae, which makes it directly different from *A. hermafrodita*; *A. serrata*; *A. columbae*; *A. galli*; *A. lineata*; *A. magalhaesi*; *A. numidae*; *A. orthocerca*; *A. pterophora* and *A. sergiomeirai* since they all have the same or more than 10 pairs of caudal papillae. *Ascaridia amblymoria* has 9 pairs of caudal papillae but differs from the new taxon by the presence of an odd papilla on the upper lip, a feature absent in *Ascaridia pavo* n. sp. Additionally, *A. hermaphrodita*, *A. galli*, *A. columbae*, *A. magalhaesi*, *A. ornata*, *A. sergiomeirai* have cervical alae, which does not occur in the new species.

*Ascaridia pavo* n. sp. has large spicules (1−3 mm), compared to *A. serrata*, *A. pintoi*, *A. pterophora* and *A. sergiomeirai*, which have spicules smaller than 1 mm (0.86−0.93 mm, 0.14 mm, 0.72−0.86 mm and 0.90−0.94 mm respectively). *Ascaridia columbae*, *A. hermaphrodita*, *A. ornata* and *A. sergiomeirai* have spicular alae, a feature that is absent in the new species.

The males of the new species have a smaller body size (8−12mm), which distinguishes them different from the others. As an example, *A. serrata* and *A. galli* are 5 to 6 times larger than *Ascaridia pavo* n. sp. Morphometric comparisons between the descriptions of *Ascaridia pavo* n. sp. are shown in Table 1, including the additional data presented in this study. Morphologically, there are few studies characterizing *Ascaridia* species, and the pathogenicity to the host (Ramos et al., 2018; Mubarokah et al., 2019; Torres et al., 2019; AL-Quraishi et al., 2020; Shohana et al., 2023).

**Table 1:** Comparison of morphometric characteristics of males *Ascaridia pavo* n. sp. with other species of *Ascaridia* in Brazil. (dimensions in mm).

**Caracteres**	***Ascaridia pavo* n. sp.**	***A. magalhaesi***	***A.*** ***pintoi***	***A.*** ***galli***	***A. lineata***	***A.*** ***hermafrodita***
	**Male**	**Male**	**Male**	**Male**	**Male**	**Male**
**Length** a	**8−12**	30	28	30−80	55−68	26−46
**Width**	**0.353−0.740**	1.46	0.7	0.971–1.26^c^	0.90	1.2−1.4
**Cervical ala**	**Absent**	Present	Not determined	Present	Not determined	Present
**Muscular esophagus (length)**	**1−2**	−	1.38	1.67–3.26^c^	−	1.5−2.1
**Number of pairs of caudal papillae**	**9**	12	9	10	10	15−16
**Precloacal papillae^b^**	**3ve**	5	−	3^c^	3^d^	5
**Adcloacal papillae^b^**	**1ve**	4	−	1ve^c^	−	1−2
**Postcloacal papillae^b^**	**5 (1ve+1sl+3L)**	3	−	6^c^(1sv+2ve+3L)	7^e^	7−8
**Spicule (length)**	**1−3**	1.66	0.14	4	1.6−2.4	2.30−2.93
**Spicular alae**	**Absent**	Not determined	Not determined	Absent	Not determined	Present
**Reference**	**Present study**	Travassos (1913)	Travassos (1933)	Cram (1927) Zhao et al. (2016)	Cram (1927)	Travassos (1930)
**Caracteres**	***Ascaridia pavo* n. sp.**	***A. serrata***	***A. amblymoria***	***A.*** ***orthocerca***	***A. numidae***	***A. columbae***
	**Male**	**Male**	**Male**	**Male**	**Male**	**Male**
**Length** a	**8−22**	46−60	40	30−40	19.4−35	16−31
**Width**	**0.353−0.740**	1.21−1.22	1.25	1−2	0.72−0.88	1.1
**Cervical ala**	**Absent**	Present	Not determined	Not determined	Not determined	Present
**Muscular esophagus (length)**	**1−2**	2.37−2.63	−	−	−	−
**Number of pairs of caudal papillae**	**9**	10	9	11	10	12−13^f^
**Precloacal papillae** b	**3ve**	3	3+1u	5	3	5^f^−7
**Adcloacal papillae** b	**1ve**	1	−	−	−	−
**Postcloacal papillae** b	**5 (1ve+1sl+3L)**	6	6	6	7	5−8^f^
**Spicule (length)**	**1−3**	0.86−0.93	−	−	3	1.2−1.9
**Spicular alae**	**Absent**	Not determined	Not determined	Not determined	Not determined	Present
**Reference**	**Present study**	Proença (1937)	Vicente et al. (1995)			Travassos (1913)
**Caracteres**	***Ascaridia pavo* n. sp.**	***A. sergiomeirai***			***A. ornata***	***A. pterophora***
	**Male**	**Male**			**Male**	**Male**
**Length^a^**	**8−12**	27−30			27−31	30.45−36.30
**Width**	**0.353−0.740**	0.60−0.68			0.69−0.80	0.59−0.72
**Cervical ala**	**Absent**	Present			Present	Not determined
**Muscular esophagus (length)**	**1−2**	0.7−1.9			−	2.27−2.83
**Number of pairs of caudal papillae**	**9**	10			9	12
**Precloacal papillae** b	**3ve**	7			−	5
**Adcloacal papillae** b	**1ve**	−			−	−
**Postcloacal papillae^b^**	**5 (1ve+1sl+3L)**	3			−	7
**Spicule (length)**	**1−3**	0.90−0.94			1.93−2.73	0.72−0.86
**Spicular alae**	**Absent**	Present			Present	Not determined
**Reference**	**Present study**	Pereira (1933)			Kajerova et al. (2004)	Christofaro & Feijó (1976)

a Measurements in millimeters unless indicate;

b Abbreviations: ve= ventral papillae, sl= sublateral papillae, L= lateral papillae, 1ph = pairs of phasmids, u= unpaired papillae;

c basede on Zhao et al. (2016);

d basede on Travassos (1913);

e basede on Pinto et al. (1991);

f basede on Santos et al. (2025).

The new species adds to the biodiversity of *Ascaridia* parasites of birds in the Phasianidae family. Zhao et al. (2016), recorded parasitism by *A. galli* in *Pavo muticus* in Xuanwuhu Zoo, Nanjing, Jiangsu Province, China. Yamaguti (1961) recorded the occurrence of *A. galli* in *Phasianus colchicus formosanus* in Japan. Additionally, this species has been recorded in different countries parasitizing *Gallus gallus* (Ackert 1931; Mozgovoi 1953; Ramadan & Znada, 1992). In Brazil, Teixeira et al. (2012) and Ramos et al. (2018) recorded the occurrence of *A. galli* in *Pavo cristatus* in the states of Bahia and Goiás respectively, using morphological and macroscopic analyses and clinical signs of the animals for diagnosis. Adding to the records of *Ascaridia* in the national territory, Vicente et al. (1995) recorded *A. columbae*, *A. galli* and *A. lineata* parasitizing *Gallus gallus* var. *domesticus* (Linnaeus, 1758) in the states of Rio de Janeiro, Rondônia, Bahia, Ceará, Maranhão, Mato Grosso, Mato Grosso do Sul, Minas Gerais, Pará, Paraná, Pernambuco, Piauí, Rio Grande do Sul, São Paulo, Santa Catarina and the Federal District and *A. galli* in *Meleagris gallopavo* Linnaeus, 1758 in Goiás, Paraná, Pernambuco and the Federal District. The same authors recorded *A. hermaphrodita*, *A. ornata* and *A. columbae* parasitizing birds in the states of Pará and Amapá, the same biogeographical region as the new taxon.
